# Supplementary figures and images for: CGRP receptor-expressing neurons in the central amygdala contributes to injury-induced pain hypersensitivity
Source: bioRxiv. 2026 Apr 6:2026.04.02.716115. Preprint. [Version 1] doi: 10.64898/2026.04.02.716115 (PMC13082058; doi:10.64898/2026.04.02.716115)

Supplementary Figure 1

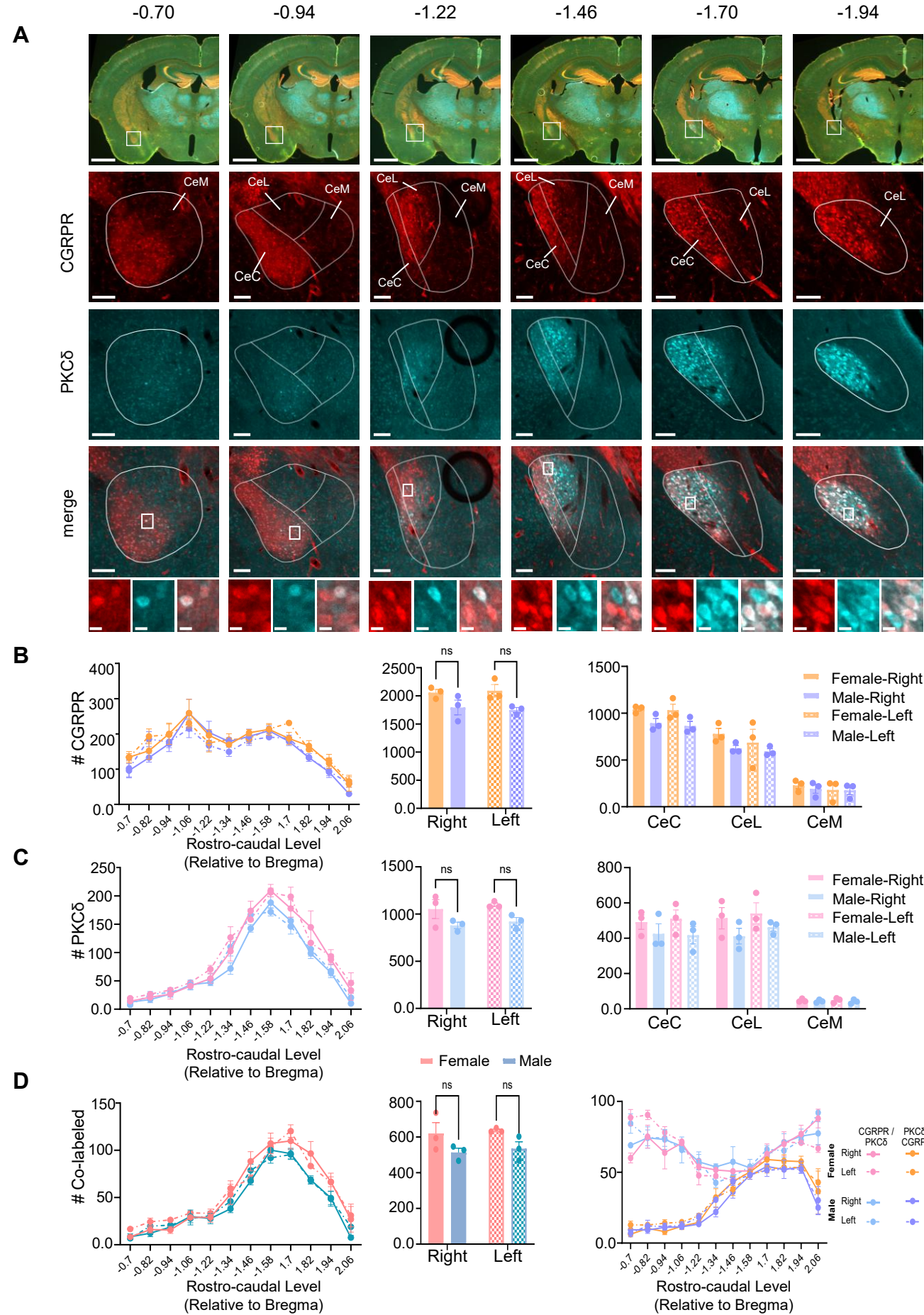

Supplement: Supplement 1 — Supplementary Figure 1: Expression of CGRPR and PKCδ in the CeA is Consistent Between Sexes and Hemispheres. (A). Representative images of coronal sections containing the CeA expressing CGRPR tdTomato (red) and immunostained for PKCδ (cyan). Scale bars represent 1000 μm in the top row, 100 μm in the next three rows, and 10 μm in bottom row. (B). Quantification of cells positive for CGRPR. Left panel – rostrocaudal level distribution showing average number of positive cells for levels −0.70 to −2.06 (n = 4 male mice, 3 female mice, 10 to 12 slices per mouse). Middle panel – average number of positive cells in entire CeA (n = 3 male mice, 3 female mice, 11 slices per mouse). Right panel – average number of positive cells per subnuclei region (n = 3 male mice, 3 female mice, 11 slices per mouse). (C). Quantification of cells positive for PKCδ. Left panel – rostrocaudal level distribution showing average number of positive cells for levels −0.70 to −2.06 (n = 4 male mice, 3 female mice, 10 to 12 slices per mouse). Middle panel – average number of positive cells in entire CeA (n = 3 male mice, 3 female mice, 11 slices per mouse). Right panel – average number of positive cells per subnuclei region (n = 3 male mice, 3 female mice, 11 slices per mouse). (D). Quantification of cells co-labeled for CGRPR and PKCδ. Left panel – rostrocaudual levels distribution showing average number of positive cells for levels −0.70 to −2.06 (n = 4 male mice, 3 female mice, 10 to 12 slices per mouse). Middle panel – average number of positive cells in entire CeA (n = 3 male mice, 3 female mice, 11 slices per mouse). Right panel – rostrocaudal level distribution of percentage of co-labeled cells, levels −0.70 to −2.06 (n = 4 male mice, 3 female mice, 10 to 12 slices per mouse). [file media-1.pdf]
